# mRNA vaccine development for cholangiocarcinoma: a precise pipeline

**DOI:** 10.1186/s40779-022-00399-8

**Published:** 2022-07-12

**Authors:** Tian-Yu Tang, Xing Huang, Gang Zhang, Ming-Hao Lu, Ting-Bo Liang

**Affiliations:** 1 Department of Hepatobiliary and Pancreatic Surgery, the First Affiliated Hospital, Zhejiang University School of Medicine, Hangzhou, 310003 China; 2 Zhejiang Provincial Key Laboratory of Pancreatic Disease, the First Affiliated Hospital, Zhejiang University School of Medicine, Hangzhou, 310009 China; 3 Zhejiang Clinical Research Center of Hepatobiliary and Pancreatic Diseases, Hangzhou, 310003 China; 4 Innovation Center for the Study of Pancreatic Diseases of Zhejiang Province, Hangzhou, 310009 China; 5 Cancer Center, Zhejiang University, Hangzhou, 310058 China

**Keywords:** Cholangiocarcinoma (CHOL), mRNA vaccine, Tumor antigen, Immune subtype, Immune microenvironment

## Abstract

Cholangiocarcinoma (CHOL) is one of the most aggressive tumors worldwide and cannot be effectively treated by conventional and novel treatments, including immune checkpoint blockade therapy. The mRNA vaccine-based immunotherapeutic strategy has attracted much attention for various diseases, however, its application in CHOL is limited due to the thoughtlessness in the integration of vaccine design and patient selection. A recent study established an integrated path for identifying potent CHOL antigens for mRNA vaccine development and a precise stratification for identifying CHOL patients who can benefit from the mRNA vaccines. In spite of a promising prospect, further investigations should identify immunogenic antigens and onco-immunological characteristics of CHOL to guide the clinical application of CHOL mRNA vaccines in the future.

## Background

Cholangiocarcinoma (CHOL) is the second most common hepatobiliary cancer and one of the most aggressive tumors worldwide [1]. Surgical resection can be curative for some CHOL patients who present with early-stage disease [1, 2]. However, CHOL is mostly detected at an advanced stage, and thus most patients are late for surgery [3]. Besides, most patients with unresectable CHOL get little benefit from systematic treatments. For instance, gemcitabine and cisplatin, as first-line treatments for CHOL, can only achieve an overall survival of less than 1 year [4]. Although fibroblast growth factor receptor and isocitrate dehydrogenase mutations-targeted drugs, as second-line treatments, can achieve a 30–35% response rate, most CHOL patients do not have such mutations and are thus not suitable for such targeted therapies [5–7]. Additionally, mitogen-activated protein kinase inhibitor selumetinib shows poor efficacy in patients with metastatic biliary cancer [8], while hepatocyte growth factor receptor inhibitor tivantinib combined with gemcitabine has a partial response in only 20% of patients [9].

## Current dilemma in CHOL immunotherapy

Since immune system plays a critical role in recognition and elimination of malignant cells, intensive studies have been conducted to seek effective approaches for activating anti-tumor immune response in the past 2 decades [10]. The immune checkpoint proteins, including programmed death (ligand) 1 [PD-(L)1] and cytotoxic T lymphocyte-associated antigen 4 (CTLA-4), help tumor cells escape from immune surveillance, and thus immune checkpoint blockade (ICB) therapy targeting such proteins has been approved to treat a wide range of tumors [10–12]. Although ICB has achieved great success in cancer immunotherapy in recent years, it has limited efficacy in CHOL [13, 14]. In KEYNOTE­028, 24 CHOL patients were enrolled in a basket trial involving PD-L1 positive solid tumor and treated with anti-PD-1 antibody pembrolizumab, of which only 4 patients (17%) achieved a partial response. Furthermore, the response rate to ICB was even lower in non-mis-match repair (MMR) deficient CHOL, although the data were mixed [15]. In KEYNOTE-158, a response rate of only 5.8% was detected among 104 patients without known MMR deficiency, with a progression-free survival of less than 2 months [7, 15]. Although the response rate of patients with MMR deficiency to ICB exceeded 40%, such MMR deficiency cases only occur in 5% of CHOL patients [3, 7]. Previous studies have demonstrated that tumor response to immunotherapy largely depends on the expression levels of tumor-specific antigens that mediate immune recognition on tumors and promote activation of anti-tumor immunity [16–18]. Tumor cells escape immune surveillance by reducing the expression of immunogenic antigens or modifying certain antigens through multiple mechanisms, while lack of tumor-specific antigen stimulation can lead to T-cell exhaustion, eventually resulting in primary and acquired tumor resistance to immunotherapy [19–23].

## Tumor vaccines for CHOL treatment

Tumor vaccines activating anti-tumor immunity with immunogenic tumor-specific or nonspecific antigens are emerging therapeutic strategies for CHOL [23, 24]. Several vaccine-based strategies, including carcinoembryonic antigen RNA-pulsed dendritic cell (DC) and immunogenic peptides plus gemcitabine, have been developed for CHOL treatment, singly or in combination (Table 1) [25–27]. The mRNA vaccine has attracted much attention in the past decades since it can stimulate immune activation through multiple mechanisms [28]. Conventionally, mRNA vaccines can be processed as normal endogenous mRNAs in antigen-presenting cells (APCs), inducing antigen expression and presentation on major histocompatibility complex class I (MHC-I) for CD8^+^ T cell activation and major histocompatibility complex class II (MHC-II) for CD4^+^ T cell activation [14, 24]. Moreover, mRNA can activate immune systems via Toll-like receptors (TLR3, TLR7, and TLR8) located on the plasma membrane or endosome of APCs, leading to cytokine production and providing an essential costimulatory signal for the activation of T and B cells [29, 30]. Additionally, mRNA can upregulate chemokine production in non-immune cells through sensing by RIG-I-like receptors, thus recruiting APCs and other immune cells to the injection sites [31, 32]. Notably, mRNA vaccines have more advantages than previously studied vaccine models for CHOL. First, mRNA vaccines can be produced on a large scale within a short period due to their mature manufacturing and cost-effective process [24, 33]. Second, mRNA vaccines can mediate the transient expression of the selected antigens without the risk of being integrated into the host genome and thus are much safer than DNA and viral vaccines [24, 34]. Third, mRNA vaccines can relatively alleviate adverse effects due to the short-term expression of encoded peptides [33, 34]. Finally, the mRNA-based techniques allow the development of a personalized treatment based on individual sequencing data of tumor samples [24, 33]. As a result, the mRNA vaccines may become the next hotspot in cancer immunotherapy (Table 2) [35–42].

**Table 1:** CHOL vaccines in clinical trials

Vaccine	Form	Combination	Indication	*n*	Clinical trial	Status
Prevnar	Pneumococcal 13-valent conjugate vaccine	Therapeutic autologous dendritic cells, external beam radiation therapy	Unresectable HCC and unresectable ICC	26	NCT03942328	Recruiting
Recombinant fowlpox-CEA (6D)/TRICOM vaccine	Fowlpox	Sargramostim	Advanced stage tumors including CHOL	48	NCT00028496	Completed
Wilms’ tumor 1 antigen	DC vaccine	Gemcitabine	Advanced stage tumors including CHOL	11	UMIN000004063 [25]	Completed
Autologous tumor lysate	DC vaccine	–	Resectable intrahepatic cholangiocarcinoma	62	UMIN000005820 [26]	Completed
CEA RNA-pulsed DC tumor vaccine	DC vaccine	–	Advanced stage tumors including extrahepatic bile duct cancer	24	NCT00004604	Completed
Amph modified KRAS peptides	Peptide	Amph-CpG-7909	Advanced stage tumors including CHOL	18	NCT04853017	Recruiting

*CHOL* cholangiocarcinoma, *HCC* hepatocellular carcinoma, *ICC* intrahepatic cholangiocarcinoma, *TRICOM* triad of costimulatory molecules, *DC* dendritic cell, *CEA* carcinoembryonic antigen, *KRAS* kirsten rat sarcoma viral oncogene, “–” no combined drug

**Table 2:** Early phase clinical studies evaluating the role of mRNA vaccines for non-CHOL cancers

Antigen	Vehicle	Combination	Indication	*n*	Outcomes	Clinical trial
WT1	DC	Durvalumab	Solid tumor Lymphoma	264	Increased WT1-specific CD8^+^ T cells	NCT03739931 [35]
WT1, PRAME, and CMVpp65	DC	–	Acute myeloid leukemia	13	Enhanced PRAME and WT1-specific immunity; 5 patients in CR, with an observation period of up to 840 d	NCT01734304
CEA-peptide	LNP	Oxaliplatin/Capecitabine	Colorectal cancer	30	CEA peptide-specific T-cells detected in 8/11 patients in the peptide group	NCT00228189 [36]
Tumor RNA plus synthetic CD40L RNA	DC	Sunitinib	Renal cell carcinoma	25	13 patients (62%) experienced clinical benefit (PR + CR)	NCT00678119 [37]
Tumor-associated antigens	Liposome	PD-1 inhibitor	Melanoma	119	Increased antigen-specific cytotoxic T-cell were observed	NCT02410733 [38]
Autologous tumor-mRNA	DC	IL-2	Melanoma	31	Antigen-specific immune response demonstrated in 51.6% patients; immune responders had better survival (median 14 months vs. 6 months, *P* = 0.030)	NCT01278940 [39]
CD40 ligand TLR4, gp100 and tyrosinase	DC	–	Melanoma	28	1 PR and 2 SD observed in 8 patients	NCT01530698 [40]
MageA3, MageA1, Melan-A, Tyrosinase, Survivin, and gp100	Protamine-protected	GM-CSF s.c	Melanoma	20	Antigen-specific T cells detected in 2/4 evaluable patients; 1 CR observed in 7 patients	NCT00204607 [41]
hTERT	DC	–	Acute myeloid leukemia	21	11 patients (58%) developed hTERT-specific T-cell responses	NCT00510133 [42]

*WT1* Wilms’ tumor 1 antigen, *DC* dendritic cell, *CMV* cytomegalovirus, *PRAME* preferentially expressed antigen in melanoma, *CR* complete response, *CEA* carcinoembryonic antigen, *LNP* lipid nanoparticle, *RNA* ribonucleic acid, *PR* partial response, *PD-1* programmed death-1, *IL-2* interleukin-2, *TLR4* toll-likereceptor4, *gp100* glycoprotein 100, *SD* stable disease, *MageA* melanoma-associated antigen, *GM-CSF* granulocyte–macrophage colony-stimulating factor, *s.c.* subcutaneous injection, *hTERT* human telomerase reverse transcriptase, “–” no combined drug

## An integrated path to identify potent CHOL antigens for mRNA vaccine development

The precise identification of most immunogenic stimulators as potent candidates for optimal immune response and therapeutic efficacy is one of the critical steps for mRNA vaccine development. Previous studies have demonstrated that bioinformatics can help identify therapeutic targets and antigens and help understand tumor-host interplay from an immune perspective. cBioPortal for Cancer Genomics, Gene Expression Profiling Interactive Analysis (GEPIA), and Tumor Immune Estimation Resource (TIMER) are well-established, easily accessible databases that largely lower the threshold of bioinformatics analysis. Particularly, the cBioPortal for Cancer Genomics is an online tool used to analyze the raw data from large-scale genomic projects, including The Cancer Genome Atlas (TCGA) and the International Cancer Genome Consortium (ICGC) [43]. GEPIA is an online resource integrating RNA sequencing data from both tumors and normal samples of the TCGA and the Genotype-Tissue Expression (GTEx) programs [44]. TIMER is an online tool for immune infiltration analysis across discrete cancers [45]. Based on these online databases and bioinformatic approaches, a new pipeline has been established to identify promising antigens for mRNA CHOL vaccine development [46]. Briefly, 1391 genes with both amplified copy numbers and mutations in CHOL were first determined as potential candidates. Subsequently, the prognostic values of the amplified and mutated genes were analyzed. Fifteen candidates were identified as closely associated with overall survival, of which 3 were also correlated with progression-free survival. Elevated mRNA levels of the three candidates [transformation/transcription domain associated protein (TRRAP), fragment crystallizable (Fc) fragment of IgG receptor 1A (FCGR1A), and CD247] were significantly correlated with a better prognosis of CHOL. Moreover, the elevated expression levels of FCGR1A, TRRAP, and CD247 were closely associated with the infiltration of DCs, macrophages, and B cells. As a result, the three antigens were identified as potential candidates for the development of CHOL mRNA vaccine, boosting anti-tumor immunity after delivery to the adapted immune system.

## A precise stratification for the identification of suitable CHOL patients for mRNA vaccine

Identifying suitable patients with the highest probability of benefiting from the treatment is also crucial for the mRNA vaccine development process [47]. Mounting evidence indicates that the distinction of tumor immune microenvironment largely determines the immunotherapeutic efficacy in different cancer patients [22]. As a result, the CHOL patients have recently been stratified into two distinct immune subtypes (ISs) with the maximum intergroup and the minimum intragroup variances (IS1 and IS2, respectively) based on the gene profiles extracted from TCGA and Gene Expression Omnibus (GEO) [46]. ISs are closely correlated with the prognosis of CHOL patients. Moreover, genes associated with immunogenic cell death and immune checkpoint are differentially expressed in the two ISs. The immune cell components in these two ISs have also been analyzed. IS1 has an immune “hot” phenotype with increased scores of activated CD8 T cells, memory T cells, and immune-suppressive cell subsets [Myeloid-derived suppressor cell (MDSC), Macrophage, and Treg]. In contrast, IS2 has an immune “cold” phenotype with decreased immune cell infiltration. The molecular signature analysis has further confirmed the immune characteristics of the two ISs, indicating that IS2 patients may benefit from mRNA vaccine for immune status alteration. The intergroup heterogeneity of ISs has been visualized through the construction of the immune landscape of CHOL, where the two ISs were further divided into smaller subgroups with distinct prognoses, thus providing valuable information for patient selection for mRNA vaccine treatment. Finally, the immune gene co-expression modules have been analyzed to identify immune hub for selecting suitable CHOL patients for treatment with mRNA vaccines [22].

## Conclusions

The novel pipeline collectively provides a practical approach for the development of an mRNA vaccine for CHOL, particularly in selecting potent antigens and appropriate patients. Notably, although treatment with mRNA vaccines is promising, mRNA vaccines for CHOL patients are still in the initial stages and may face several challenges in the future. First, an in silico study has identified potential CHOL antigens based on integrated information of expression, mutation, prognostic value, and correlation with infiltrated immune cells, however, it is difficult to estimate whether such candidates can induce anti-CHOL immunity in vivo. Moreover, at advanced stages, CHOL may have already developed numerous mechanisms for immune escape, eventually causing therapeutic resistance. Additionally, the requirement for activation of anti-tumor immunity is highly variable among different patients due to CHOL heterogeneity. Last but not least, the required dosage of mRNA vaccine to treat tumors is usually higher than prophylactic vaccination for infectious diseases, which may raise concerns about the safety of mRNA vaccines in CHOL patients. Therefore, advanced investigations focusing on understanding the onco-immunological characteristics of CHOL, especially the intra-tumoral heterogeneity, tumor-microenvironmental crosstalk, and microenvironmental instability, are needed for precise and feasible application of CHOL mRNA vaccine in clinics, improvement of its effectiveness and safety, as well as the prognosis of CHOL patients.

## Data Availability

Data sharing is not applicable to this article as no datasets were generated or analyzed during the current study.

## Abbreviations

APC: Antigen-presenting cell
CHOL: Cholangiocarcinoma
CTLA-4: Cytotoxic T-lymphocyte-associated protein 4
DC: Dendritic cell
GEO: Gene expression omnibus
GEPIA: Gene expression profiling interactive analysis
GTEx: Genotype-tissue expression
ICB: Immune checkpoint blockade
ICGC: International cancer genome consortium
IS: Immune subtype
MDSC: Myeloid-derived suppressor cell
MHC-I: Major histocompatibility complex class I
MHC-II: Major histocompatibility complex class II
MMR: Mis-match repair
PD-(L)1: Programmed death (ligand)-1
TCGA: The cancer genome atlas
TLR: Toll-like receptor
TIMER: Tumor immune estimation resource
